# Predictors of fatigue in Individuals With Late-Life Depression

**DOI:** 10.1016/j.osep.2025.08.003

**Published:** 2025-09-06

**Authors:** Amanda Sce, Breno S. Diniz

**Affiliations:** UConn Center on Aging (AS, BSD), University of Connecticut Health Center, Farmington, CT.

**Keywords:** Disability, fatigue, late-life depression

## Abstract

**Objectives:**

To examine fatigue levels in individuals with late-life depression (LLD) and to identify which specific factors significantly affect fatigue severity in these individuals.

**Methods:**

We included 91 adults (51 LLD and 40 nondepressed individuals), 60% femeles, age range between 60 and 85 yeas. Diagnosis of major depressive episode was defined by the DSM-5 diagnostic criteria and fatigue severity was evaluated by the Fatigue Assessment Scale.

**Results:**

Individuals with LLD showed significantly higher total scores on the FAS (t_90_=11.81, p=0.002). Fatigue severity was signifcanlty predicted by MADRS (t_82_ = 2.74, p = 0.07), PSS (t_82_ = −4.21, p <0.001), and WHODAS scores (t_82_ = 5.23, p <0.001).

**Conclusion:**

Fatigue is common and disabling symptom associated with LLD. Interventions that promote reduction in fatigue levels can have a significant impact on disability and quality of life of individuals with LLD.

## OBJECTIVE

Fatigue can be described as a state of lessened energy due to excessive or prolonged exposure to stressors. Fatigue can be due to a primary symptom (e.g., chronic fatigue syndrome) or secondary to other psychiatric or physical illnesses and can be viewed as a condition of chronic exhaustion. Fatigue can be further divided into physical or mental components. Physical fatigue can be marked by feelings of being tired, and lower energy and activity levels. Mental fatigue is marked more by the inability to motivate to do tasks or issues concentrating on tasks. Higher fatigue levels are associated with greater levels of disability.

Late-life depression (LLD) is common and affects approximately 7% of community-dwelling older adults.^1^ LLD is characterized by the loss of interest and or motivation to participate in activities in individuals over 60 years old. Other symptoms include decreased appetite and physical activity, isolation from social activities and environments, persistent negative feelings or thoughts, disruption of normal sleep patterns, increased agitation or irritability, trouble concentrating, and an increase in harmful habits, including drinking and smoking. Fatigue is a prominent symptom of depression and disability. On the other hand, fatigability can be an indicator of underlying sickness. Fatigue is a common symptom, yet there appears to be a lack of research on the topic of fatigue and what specifically causes an individual to feel greater levels of fatigue.

There is a large overlap in fatigue and depressive symptoms in LLD and It is important to understand what specifically increases or decreases fatigue levels in these individuals. Therefore, this study aims to investigate the independent impact of LLD on fatigue levels (including its physical and mental components) and which are the variables that are associated with it in these individuals.

## METHODS

### Sample and Assessments

We included 91 participants (51 with LLD and 40 never-depressed comparison individuals), 60% of whom were female, with an age range of 60−86 years. The diagnosis of a major depressive episode was based on the DSM-5 criteria. Exclusion criteria were prior diagnosis of bipolar disorder, schizophrenia, or other psychotic disorder, history of alcohol or drug abuse in the past 6 months (except tobacco), current dementia diagnosis, or being medically unstable. Patients under antidepressant treatment were included if the medication was stable for at least 1 month.

Fatigue symptoms were evaluated by the Fatigue Assessment Scale (FAS).^2^ This scale evaluates two fatigue domains, physical and mental, and its scores range from 10 to 50, with higher scores indicating higher fatigue complaints. The severity of the depressive symptoms was assessed by the Montgomery-Asberg Depression Rating Scale (MADRS),^3^ and the severity of anxiety symptoms by the GAD-7.^4^Perceived stress was evaluated by the Perceived Stress Scale (PSS)^5^ and mental illness-related disability by the WHODAS v.2, 12 items scale.^6^ The medical comorbidity burden was evaluated using the Cumulative Illness Rating Scale, geriatric version (CIRS-G).^7^ Season was defined based on the date of the research assessment.

### Statistical Analysis

Pearson correlation analyses were done to investigate the association between fatigue levels, depressive symptoms, other demographic and clinical variables in the whole sample. Chi-square test were done to evaluate for difference in the sex distribution between diagnosis groups. Next, we used t-test to evaluate the unadjusted mean difference in other demographic and clinical variables between LLD and never-depressed comparison groups. Analysis of covariance were further carried out to evaluate the impact of confounders on the association between diagnosis and fatigue levels. Finally, we tested which are the strongest predictors of fatigue levels in whole sample and in each diagnostic group using multiple linear regression with Backward variable selection method with a removal criterion of F > 0.1.

## RESULTS

Clinical and sociodemographic characteristics of the sample is shown in Table 1. Individuals with LLD were younger, less educated, and showed higher fatigue levels, greater perceived stress, disability, higher loneliness levels, and higher BMI. As hypothesized, individuals with LLD showed significantly higher total scores on the FAS (t_90_ = 11.81, p = 0.002) and higher scores on the mental (t = 11.935, p = <0.001) and physical fatigue (t_90_= 9.92, p= 0.002) subcomponents of this scale. The association between LLD and fatigue remained statistically significant (F(1,89) = 4.08, p = 0.04) after controlling for the potential effect of confounding variables (perceived stress, age, systemic illness burden, loneliness, and disability).

Next, we evaluate the predictors of fatigue levels in the whole sample. We found that total MADRS (t_82_ = 2.74, p = 0.007), PSS (t_82_ = −4.21, p <0.001), and WHODAS scores (t_82_ = 5.23, p <0.001) were the strongest predictors of FAS scores in the whole sample. Mental fatigue was significantly predicted by MADRS (t_82_ = 3.02, p = 0.03), PSS (t_82_ = 3.56, p <0.001), WHODAS scores (t_82_ = 2.75, p = 0.007). Physical fatigue was significantly predicted by PSS scores (t_82_ = 5.03, p <0.001) and disability (t_82_ = 5.85, p <0.001).

Since fatigue perception can be affected by seasonal changes, we finally evaluated if the Seasons of depressive symptoms assessment affected the fatigue levels. We did not find any significant impact of different Seasons on fatigue level in the whole sample or when analyzing each diagnostic group individually.

## CONCLUSIONS

The current study evaluated the association between fatigue and its specific components (physical and mental fatigue) with LLD. As expected, individuals with LLD showed higher fatigue levels compared to never-depressed individuals, even after controlling for potentially confounding variables. The strongest predictors of fatigue in the whole sample, beyond the severity of depressive symptoms, were perceived stress and disability. A similar pattern emerged after splitting the sample into LLD and never-depressed groups. Interestingly, the season of the year did not significantly influence fatigue levels in LLD or never-depressed individuals.

Although fatigue is a well-known symptom of depression and part of its diagnostic criteria, there is little research about its most important predictors in older adults with LLD. Total fatigue levels in our study were strongly associated with disability, measured by the WHODAS v2 scale, and perceived stress. Our findings are aligned with recent studies showing a correlation between fatigue, disability, and perceived stress.^8^ The mechanisms for these associations may be manyfold. For example, prolonged exposure to stress may contribute to physiological (e.g., sympathetic system hyperactivation, increase in inflammatory burden, hypothalamic-adrenal axis dysregulation) and behavioral changes (e.g., sleep pattern disruption) that can lead to greater fatigue levels. In turn, greater fatigue can worsen common depressive symptoms, like poor concentration and motivation) leading to greater disability related to a depressive episode.

We hypothesized that fatigue levels would be influenced by seasonality as observed in some studies in the literature as expected as depressive symptoms can fluctuate according the season of the year^9^ (PMID 26846791). However, we found no significant effect of the season of the year on fatigue in this sample. Our results are in line with prior studies in other medical conditions (e.g., rheumatoid arthritis and Sjögren’s syndrome)^10,11^ and suggest that seasonality is not a relevant variable when evaluating fatigue in LLD. Alternatively, we did not include individuals with seasonal affective disorder, a condition in which depressive symptoms are more intense or manifest mostly during the winter. Thus, we cannot exclude the possibility that fatigue levels would be impacted by the season of the year given the high correlation between fatigue and depressive symptoms.

Based on previous studies, we expected that seasonality would affect levels of fatigue, specifically in the fall and winter. However, our results did not support this hypothesis, since we found no significant impact of seasons on fatigue levels. Our results are in line with previous evidence that found no changes in chronic fatigue symptoms due to seasonal changes.^12^ Other possible explanations for our null findings is that we did not include participants seasonal affective disorders, where the depressive symptoms, and possibly fatigue levels, are more intense during the winter. Future studies, including individuals with seasonal affective disorder, would be more suitable to address the impact of seasonality on fatigue levels.

Fatigue is part of the diagnostic criteria for major depressive episodes, and, as expected, showed a strong correlation with the severity of global depressive symptoms in our sample. However, previous studies have found that while treatment for depression may alleviate depressive symptoms, residual fatigue still remains an issue,^13^ since it can increase the risk of recurrence and long-term functional disability.^14^ Currently, there exists little research on treatments targeting fatigue in LLD. Nonpharmacological interventions, such as physical activity, or pharmacological interventions, such as use of noradrenergic stimulants, one example being modafinil, can help improve fatigue, though such evidence is usually derived from small clinical trials or observational studies.

Our results should be viewed in light of the study limitations. First, this is a cross-sectional analysis and we cannot infer causal relationships between fatigue, disability and perceived stress. Also, the LLD participants were derived from a clinical sample, and lacked racial and ethnic diversity. Thus, the results may not be generalizable to the general population. Sleep disturbances are usually associated with fatigue in depressed and nondepressed individuals. However, we did not specifically evaluate sleep disturbances in this sample and we cannot rule out their influence on our findings. Also, the ascertainement of seasonality was based on the date of psychiatric assessment. Therefore, we might have not captured a more nunaced effect fo seasonality on fatigue levels in this sample. Therefore, future studies, including larger and more diverse sample sizes, are necessary to address the mechanisms linking fatigue, perceived stress and disability in LLD.

In conclusion, our study showed that individuals with LLD experienced significantly more intense fatigue than nondepressed individuals. Given the importance of fatigue in the management of depressive episodes, future studies should focus on specific management strategies for improving it among individuals with LLD.

## Figures and Tables

**TABLE 1.** Demographic and Clinical Characteristics of the Sample

		Diagnosis		Statistics	d.f.	p-Value
		LLD	NDC			
Age (years)		68 ± 6	72 ± 8	T = 2.58	89	0.012
Sex	Male	33.3%	47.5%	X^2^ = 1.82	1	0.12
	Female	66.7%	52.5%			
Race	White	86.3%	70.0%	X^2^ = 3.62	2	0.16
	African descent	3.9%	7.5%			
	Asian	9.8%	22.5%			
Years of education		14 ± 2	15 ± 2	1.89	89	0.07
MADRS		18 ± 7	1 ± 2	15.70	89	<0.001
FAS (Total score)		32 ± 8	15 ± 4	11.81	89	<0.001
FAS (physical subscore)		16 ± 5	8 ± 2	9.91	89	<0.001
FAS (mental subscore)		16 ± 4	7 ± 2	11.93	89	<0.001
PSS		23 ± 7	7 ± 5	12.44	89	<0.001
WHODAS v2		31 ± 17	5 ± 6	9.15	89	<0.001
UCLA v3		32 ± 15	6 ± 7	9.88	89	<0.001
CIRS-G		10 ± 4	7 ± 5	2.85	88	0.005

## Data Availability

The data has not been previously presented orally or by poster at a scientific meeting.

## Highlights

- What is the primary question addressed by this study?
  To evaluate the impact of fatigue in late-life depression (LLD) and its related predictors in these individuals.
- What is the main finding of this study?
  Fatigue was significantly higher in individuals with LLD. Moreover, fatigue was strongly predicted by disability and perceived stress in this population.
- What is the meaning of the finding?
  Fatigue is a common feature of LLD and can be a target for intervention in these individuals.

## DATA SHARING STATEMENT

All data related to this analysis can be shared by contacting Dr. Diniz.

## References

[1] Borges MK, Romanini CV, Lima NA, : Longitudinal Association between late-life depression (LLD) and frailty: findings from a prospective cohort study (MiMiCS-FRAIL). J Nutr, Health Aging 2021; 25, 985–902 34409968 10.1007/s12603-021-1639-x PMC8103429

[2] Drent M, Lower EE, Vries JD: Sarcoidosis-associated fatigue. Eur Respirat J 2012; 40:255–263 22441750 10.1183/09031936.00002512

[3] Montgomery SA, Asberg M: A new depression scale designed to be sensitive to change. Br J Psychiatry 1979; 134:382–389 444788 10.1192/bjp.134.4.382

[4] Plummer F, Manea L, Trepel D, : Screening for anxiety disorders with the GAD-7 and GAD-2: a systematic review and diagnostic metaanalysis. Gen Hosp Psychiatry 2016; 39:24–31 26719105 10.1016/j.genhosppsych.2015.11.005

[5] Lee EH: Review of the psychometric evidence of the perceived stress scale. Asian Nurs Res 2012; 6:121–127 10.1016/j.anr.2012.08.004 25031113

[6] Üstüun TB, Kostanjsek N, Chatterji S, : Measuring health and disability: manual for World Health Organization Disability Assessment Schedule (WHODAS 2.0). World Health Organization, 2010 https://www.who.int/standards/classifications/international-classification-of-functioning-disability-and-health/who-disability-assessment-schedule

[7] Miller MD, Paradis CF, Houck PR, : Rating chronic medical illness burden in geropsychiatric practice and research: application of the Cumulative Illness Rating Scale. Psychiatry Res 1992; 41:237–248 1594710 10.1016/0165-1781(92)90005-n

[8] Corfield EC, Martin NG, Nyholt DR: Co-occurrence and symptomology of fatigue and depression. Comprehens Psychiatry 2016; 71:1–10 10.1016/j.comppsych.2016.08.004 27567301

[9] Juruena MF, Cleare AJ: Overlap between atypical depression, seasonal affective illness and chronic fatigue syndrome. Braz J Psychiatry 2007; 1:19–26 10.1590/s1516-44462007000500005 17546343

[10] Duret PM, Meyer N, Saraux A, : Seasonal effect on fatigue, pain and dryness in primary Sjögren’s syndrome. Arthritis Res Ther 2020; 22:39 32093783 10.1186/s13075-020-2118-1 PMC7041128

[11] Feldthusen C, Grimby-Ekman A, Forsblad-d’Elia H, : Seasonal variations in fatigue in persons with rheumatoid arthritis: a longitudinal study. BMC Musculoskel Disord 2016; 17:59 10.1186/s12891-016-0911-4 PMC4743196 26846791

[12] Borreguero DG, Dale JK, Rosenthal NE, : Lack of seasonal variation of symptoms in patients with chronic fatigue syndrome. Psychiatry Res 1988; 77:71–77 10.1016/s0165-1781(97)00141-8 9541142

[13] Targum ST, Fava M: Fatigue as a residual symptom of depression. Clin Neurosci 2011; 10:40–43 PMC3225130 22132370

[14] Marin H, Menza MA: Specific treatment of residual fatigue in depressed patients. Psychiatry 2004; 2:12–18 PMC3012615 21197374

